# Characterization of Lactic Acid Bacterium Exopolysaccharide, Biological, and Nutritional Evaluation of Probiotic Formulated Fermented Coconut Beverage

**DOI:** 10.1155/2024/8923217

**Published:** 2024-09-02

**Authors:** Bukola Christianah Adebayo-Tayo, Bukola Rachael Ogundele, Oladeji Aderibigbe Ajani, Olusola Ademola Olaniyi

**Affiliations:** ^1^ Department of Microbiology University of Ibadan, Ibadan, Oyo State, Nigeria; ^2^ Federal Bureau of Prisons United States Department of Justice Federal Medical Center, Old N. North Carolina HWY 75, Butner, North Carolina 27509, USA; ^3^ Department of Mathematics and Computer Science University of North Carolina, Pembroke, North Carolina, USA

**Keywords:** antioxidant, exopolysaccharide, fermented coconut beverage, nutritional, probiotic lactic acid bacteria

## Abstract

Exopolysaccharides (EPSs), produced by lactic acid bacteria (LAB), play a crucial role in enhancing the texture and stability of yoghurt by forming a protective matrix that helps to maintain its rheological and sensory characteristics. The search for a dairy alternative for the lactose-intolerant populace is a necessity, and the use of probiotic LAB and their EPS to formulate fermented coconut beverage (FFCB) will be of added advantage. The production and characterization of EPS from a LAB strain isolated from yoghurt, its probiotic and antioxidant potential, and its application in the production of probiotic FFCB were investigated. The EPS produced by the isolate was characterized using a scanning electron microscope (SEM), high-performance liquid chromatography, Fourier transform infrared (FT-IR) spectroscopy, and energy-dispersive X-ray. The antioxidant potential of the EPS was determined. The isolate probiotic potential, such as tolerance to low pH, bile salts, gastric pH, autoaggregation, coaggregation, antimicrobial potential, and antibacterial activity, was evaluated, and the isolate was identified using 16S rRNA. The LAB strain and the EPS were used for the formulation of probiotic FFCB, and the proximate mineral composition of the enriched yoghurt was determined. Isolate W3 produced 6204.50 mg/L EPS. The EPS produced by the LAB was spherical with a coarse surface. Hydroxyl, carboxyl, and *α*-pyranose were the major functional groups present in the EPS. Eight monosaccharides were present in glucose, which has the highest molar ratio. The EDX spectra ascertain the presence of carbon, oxygen (carbohydrate), and other elements. The purified EPS exhibited antioxidant activity in a dose-dependent manner. DPPH, FRAP, TAC, and TPC of the EPS ranged from 42.36% to 75.88%, 2.48 to 5.31 *μ*g/mL, 1.66 to 3.57 *μ*g/mL, and 1.42 to 2.03 *μ*g/mL, respectively. The LAB strain exhibited moderate tolerance to low pH, bile salts, gastric juice, good autoaggregation (13.33%), coaggregation (0%–59.09%) with *E. coli*, and varied sensitivity to different antibiotics used. The isolate is hemolysis, deoxyribonuclease (DNase), and lecithinase negative, possesses characteristics of probiotics, and could have the ability to confer health benefits. The LAB strain has a 100.0% pairwise identity to *Pediococcus acidilactici*. The FFCB has pH, lactic acid, specific gravity, total soluble solids (TSSs), and vitamin C content ranging from 5.81 to 6.8, 10.8 to 55.8 mg/L, 0.910 to 1.394 kg/m^3^, 0.136 to 0.196 °Bx, and 0.26% to 0.66%. The formulated beverage fermented with a commercial starter had the highest lactic acid at Day 7 of storage. The FFCB sample with the commercial starter and the probiotic strain had the highest ash and crude fiber content (1.3%, 0.68%). The FFCB fortified with EPS showed the highest protein content (4.6%). The formulated yogurt samples fortified with the highest concentration of EPS had the highest calcium content after 7 days of storage (162.31 ± 0.01^a^). In conclusion, EPS produced by *Pediococcus acidilactici* was a heteropolymeric EPS with good antioxidant activity, and the LAB strain exhibited a good starter for producing FFCB enriched with EPS. The FFCB has good nutritional characteristics and could serve as a functional and natural nutraceutical food for the lactose intolerance population.

## 1. Introduction

Exopolysaccharides (EPS) are complex carbohydrates that are produced by a variety of microorganisms, including bacteria, most especially lactic acid bacteria (LAB), archaea, and fungi [1]. Microbial EPS refers to all forms of bacterial polysaccharide, both slime and capsule, found outside the cell wall [2]. EPS produced by LAB has been known to have probiotic effects as well as therapeutic, anti-inflammatory, and immunomodulatory effects, as a natural thickener, stabilizer, emulsifier, and viscosifying agent in the food industry [3–5].

LABs are a nonpathogenic group of Gram-positive bacteria that produce lactic acid as an end product of fermentation. They are commonly used in dairy industries, most especially in cheese and yogurt production, sauerkraut, and pickles. It has the potential to change the intestinal environment, reduce lactose intolerance, and enhance the immune system [6]. Different strains of LAB that are capable of producing EPS are S*treptococcus*, *Lactococcus*, *Pediococcus*, *Lactobacillus*, *Leuconostoc*, and *Weissella* species [7]. Zaghloul and Ibrahim [8] reported the chemical composition and in vitro healing potential of EPS from probiotic *Lactiplantibacillus plantarum* EI6 isolated from a marine environment. EPSs produced by microorganisms are biodegradable and nontoxic compared to synthetic polymers [9–11]. LABs are being investigated for their potential health benefits, such as improving digestion, boosting the immune system, and preventing infections (probiotics) [12].

Probiotics are living microorganisms that, when administered in adequate amounts, confer a health benefit to the host [13]. They are a live microbial food ingredient that beneficially affects the host by improving its intestinal microbial balance [14]. Probiotics are present in fermented dairy products such as yogurt, cheese, and buttermilk. LABs are the highest source of probiotics. However, not all LABs are probiotics, and LAB should be evaluated for their probiotic attributes and safety profile.

Yoghurt is a fermented product obtained from the catabolism of milk lactose through anaerobic fermentation by some probiotic LAB [15]. Yoghurts are commonly obtained from cow milk and other substrates such as evaporated whole milk or skimmed solid milk [16]. Yoghurt, as a fermented dairy product, is regarded as a probiotic carrier with nutritional benefits beyond those of milk and can be consumed moderately by lactose-intolerant individuals without ill effects [17, 18].

As a result of the high cost of cow milk and the limitations of vegetarians and lactose intolerance in their quest for probiotic yogurt consumption, the search for alternative substrates like coconut milk will be of added advantage. Production of yoghurt using coconut milk and other milk from legumes has been reported to be delicious and nutritious [19, 20].

Coconut (*Cocos nucifera*) milk has been used as a flavor and taste enhancer in many products, such as ice cream, biscuits, and bakeries [21, 22]. Coconut milk has been reported to be rich in minerals such as calcium and Vitamin A, and the total saturated fat is above 10% of the total energy [23, 24].

The desire to have functional and natural food is on the increase. Lactose intolerance and the use of chemical additives as viscosifiers, thickeners, stabilizers, and texturing agents in yogurt production are of great concern because of the side effects. The search for a dairy alternative for the lactose-intolerant populace is a necessity. The use of coconut milk as an alternative to dairy milk, LAB, and their EPS to improve the nutritional, texture, and rheological properties of fermented coconut beverage will be of added advantage, which necessitates this study.

This study is aimed at the production and characterization of EPS from LAB, the evaluation of the probiotic potential of the isolate, and the production of fermented coconut beverage using the EPS and the probiotic LAB strain.

## 2. Materials and Methods

### 2.1. Samples and Culture Collection

Tall coconut varieties with matured fruit, commercial powdered full cream (Dano) milk, and commercial freeze-dried *yoghurt* starter (*Lactobacillus bulgaricus* and *Streptococcus thermophilus*) were obtained from Sango Market and Supermarket in Ojoo, Ibadan, Oyo State, Nigeria.

*Pediococcus acidilactici* strain was previously isolated from *Wara* (a traditional African fermented milk product, typically made by adding a coagulant to fresh milk, which curdles the milk protein and whey, resulting in a soft, jiggly, and high-protein food). Foodborne pathogens (*Escherichia coli*, *Salmonella typhimurium*, *Aeromonas hydrophila*, *Klebsiella pneumonia*, and *Bacillus subtilis*) were collected from the Department of Microbiology, University of Ibadan, Nigeria.

### 2.2. EPS Production and Quantification

*Pediococcus acidilactici* strain was used for EPS production using a modified EPS selection medium (mESM) containing 5% skimmed milk, 0.35% yeast extract, 0.35% peptone, and 5% sucrose [25]. The pure culture of the isolate from the stock culture was subculture using sterile MRS broth incubated at 37°C for 48 h. Ten milliliters of the isolate (containing 1.5 × 10^6^ CFU/mL standardized inoculum using 0.5 McFarland standards) was inoculated into the sterile mESM and incubated at 35°C for 72 h. The fermentation medium was centrifuged at 10,000 rpm for 20 min to remove the cells. The supernatant was boiled in a water bath at 90°C for 10 min to stop enzymatic degradation by phosphoglucomutase. Two hundred fifty micrograms of 80% (*w*/*v*) trichloroacetic acid was added (as a precipitating agent to precipitate the EPS and allow easy separation of the EPS) and then mixed with cold ethanol (to purify the EPS) and stored at 4°C overnight to prevent degradation process. The stored sample was centrifuged (12,000 rpm) for 15 min to recover the EPS, and the EPS was air-dried [26, 27]. The experiment was performed in triplicate. The total sugar concentration was determined using the phenol-sulfuric acid method, the EPS was expressed in milligrams per liter, and glucose was used as a standard [28].

### 2.3. Characterization of EPS

The EPS monosaccharide composition was determined using high-performance liquid chromatography (HPLC) [29]. The EPS sample was hydrolyzed using 1 mL of trifluoroacetic acid (2 M) at 120°C for 2 h. Hydrolyzed samples were derivatized using 1-phenyl-3-methyl-5-pyrazolone, and the sample was analyzed using an HPLC system containing a four-unit pump (Agilent Technologies, Wilmington, United States) and a Shim-pak VPODS column (4.6 × 150 mm). Sodium phosphate (50 mM, pH 7.0) and acetonitrile (82% and 18%) (*V*/*v*) were used as the mobile phase. The sample elution was done at a 1.0 mL/min flow rate using a refractive index detector at 245 nm. The area under the curves was measured using an integrator, and the different sugar standards were compared with the known retention times of the EPS samples. The EPS production was expressed as milligrams per liter.

Fourier transform infrared (FT-IR) spectroscopy is used to determine the functional groups present in the EPS samples. The KBr method was used to determine the spectrum of the EPS. The dried EPS samples were pressed into KBr pellets (1:100). The FT-IR spectra of the pelleted samples were measured at 4 cm^1^ in the region of 4000–400 cm^1^ using the Bruker Tensor 27 instrument [30].

Scanning electron microscopy was used to determine the surface morphological structure of the EPS samples. The purified EPS's microstructure and surface morphology were examined using a Carl Zeiss Supra 55 Gemini Scanning Electron Microscope (SEM) from German Technology Jena, Germany, operating at an accelerating voltage of 20 keV. The EPS was mounted to the metal stub and coated with a layer of gold via sputtering. Micrographs were captured at increased magnification to guarantee sharp images. The EPS's elemental composition was analyzed using an energy-dispersive X-ray analyzer, namely, the Oxford Instruments (EDX) attached to a SEM. The X-ray spectra of the elements were acquired using an accelerating voltage of 15 keV.

### 2.4. In Vitro Antioxidant Potential of the EPS

In vitro antioxidant potential of the EPS sample as determined using the 2-diphenyl-1-picyryl-hydrazyl-hydrate (DPPH) assay, ferric ion-reducing antioxidant power (FRAP), total antioxidant activity, and total phenolic (TP) content.

The DPPH radical scavenging activity of the EPS was determined using the modified method of Gülçin [31]. Two milliliters of DPPH solution (0.1 mM DPPH in 500 mM ethanol) was added to 2 mL of different concentrations (50, 200, and 1000 *μ*g/mL) of the EPS samples, respectively. The reaction mixture was incubated in the dark at room temperature for 30 min. The absorbance of the mixture was taken at 517 nm. Ethanol was used as a blank, while a DPPH solution in ethanol served as the control. A reduction in absorbance indicates DPPH radical scavenging activity. The DPPH radical scavenging activity of the samples was expressed as follows:

 $$\begin{array}{c} \%\text{inhibition of DPPH absorbance}:\text{inhibition}=\frac{\left(\text{A control}-\text{A test}\right)}{\text{A control}}\times 100 \end{array}$$

where A control is the absorbance of the control sample (DPPH solution without an EPS sample) and A test is the absorbance of the test sample (DPPH solution plus an EPS sample).

The FRAP of the EPS samples was measured according to the method of Oyaizu [32], with slight modifications by Gülçin et al. [33]. Different concentrations of the EPS sample (200–1000 *μ*g/mL) were mixed with 2.5 mL of phosphate buffer (20 mM) and 2.5 mL of potassium ferricyanide (1%). The mixture was incubated at 50°C for 30 min, and 2.5 mL of trichloroacetic acid (10%) and 0.5 mL of ferric chloride (0.1%) were added to the mixture. It was then kept for 10 min. The absorbance was taken at 700 nm, and ascorbic acid was used as a positive reference standard. All assays were done in triplicate and averaged.

The total antioxidant activity of EPS was determined using the method of Mitsuda, Yasumoto, and Iwami [34], as reported by Kanamarlapudi and Muddada [35]. A total antioxidant solution containing a mixture of 1.235 g of ammonium molybdate (4 mM), 0.6 M sulfuric acid (45 mL), 0.9942 g of sodium sulfate (28 mM), and 250 mL of distilled water was used as the total antioxidant capacity. About 0.1 mL of different concentrations (200–1000 *μ*g) of the EPS sample was added to 1 mL of total antioxidant solution, and absorbance was taken at 695 nm after 15 min of incubation. Ascorbic acid was used as a standard.

The TP contents of the EPS sample were determined spectrophotometrically [36]. In brief, 0.5 mL of each extract was made up to 3 mL of a different concentration of EPS solution and mixed with 0.5 mL of Folin–Ciocalteu's phenol reagent. After 5 min, 2 mL of Na_2_CO_3_ (2%) solution was added to the reaction mixture, mixed thoroughly, and then kept at 30°C for 60 min in a dark place. The absorbance was taken at 650 nm. The TP was determined from the extrapolation of a calibration curve constructed using standard concentrations of gallic acid solution. The estimation of phenolic compounds was carried out in triplicate. The TP was expressed as milligrams of gallic acid equivalents (GAEs) per gram of dried sample. Gallic acid was used as a standard [37].

### 2.5. Probiotic Potential of the LAB Strain

Tolerance of the isolate to an acidic environment was determined by culturing the 1 mL (containing 1.5 × 10^6^ CFU/mL standardized inocum using 0.5 McFarland standard) of the isolate in acidified MRS broth at pH 2.5, 3.0, 3.5, and 4.0 and nonacidified MRS broth at pH 6.4. for 24 h at 37°C under anaerobic conditions. The absorbance of the samples was taken using a spectrophotometer at 600 nm after 0 and 24 h of incubation. The survival rate was calculated as

 $$\begin{array}{c} \text{Survival}\%=\left(\frac{{\text{OD}}_{600}\;\left(24\text{ h}\right)}{{\text{OD}}_{600}\;\left(0\;h\right)}\right)\times 100 \end{array}$$

where OD_600_ (24 h) is the growth value of cultures at pH 2.5, 3.0, 3.5, and 4.0 and OD_600_ (0 h) is the growth value of cultures at pH 6.2.

Tolerance of the isolate to bile salt was determined by culturing the isolate in MRS containing different bile salt concentrations (0.1%, 0.3%, and 0.5% bile salt). MRS without the addition of bile salt serves as a control. The inoculated samples were incubated at 37°C for 4 h. The growth was evaluated by measuring the absorbance at 600 nm using a spectrophotometer.

 $$\begin{array}{c} \text{Bile salt tolerance was expressed as}:\text{Survival}\%=\frac{\text{Log }N1}{\text{Log }No}\times 100 \end{array}$$

Gastrointestinal transit tolerance of the LAB strain was determined by using Electrolyte A (containing NaCl, 6.2 g/L; KCl, 2.2 g/L; CaCl_2_, 0.22 g/L; NaHCO_3_, 1.2 g/L, *w*/*v*, pH 6.2) label G1, which is the control. Electrolyte A was adjusted to different pHs (4.0, 3.5, 3.0, and 2.5) labeled G2–G5 which was used for in vitro mimicry of the gastrointestinal tract. One milliliter of the LAB suspension was inoculated on the pH-adjusted G1–G5 electrolyte and incubated at 37°C for 120 min. The cell pellet, after centrifugation at 8000 rpm at 4°C for 5 min, was suspended in a sterile saline solution (0.85% NaCl) and then plated on MRS agar. The survival rate was calculated using the following equation:

 $$\begin{array}{c} \text{Survival}\%=\frac{\text{Log }N1}{\text{Log }N\text{o}}\times 100 \end{array}$$

where *N*1 is the growth value of cultures at pH 4.0, 3.5, 3.0, and 2.5 and *No* is the growth values of cultures at pH 6.2.

The autoaggregation of the isolates was determined using the method of Khalil et al. [38]. Harvested cells were washed twice with phosphate buffer solution (PBS) (pH 7.2). The cells were resuspended in PBS (McFarland standard 0.5), vortexed thoroughly, and incubated at 37°C for 5 h. Aliquots (1 mL) were taken from each sample at 0 and 5 h, and the absorbance was measured at OD 660 nm. Autoaggregation was calculated using

 $$\begin{array}{c} \frac{\text{Ao}-\text{As}}{\text{Ao}}\times 100 \end{array}$$

where As is the absorbance at 5 h of incubation and Ao is the absorbance at 0 h.

The coaggregation assay of the isolates determined using the method of Khalil et al. [38]. The suspension of the LAB strain as well as individual pathogenic bacteria (namely, *Escherichia coli*, *Salmonella typhimurium*, *Aeromonas hydrophila*, *Klebsiella pneumonia*, and *Bacillus subtilis*) cells was washed twice with PBS (pH 7.2). The cells were resuspended in PBS (McFarland standard 0.5), vortexed thoroughly, and incubated at 37°C for 5 h. Aliquots (1 mL) were taken from each sample at 0 and 5 h, and the absorbance was measured at OD 660 nm. Coaggregation was calculated using

 $$\begin{array}{c} \frac{\text{Ao}-\text{As}}{\text{Ao}}\times 100 \end{array}$$

where Ao is the absorbance of the mixed bacteria suspension at 0 h of incubation.

What is the absorbance of the mixed bacteria suspension after 5 h?

The antibiotic resistance potential of the isolate was determined using the method of Khalil et al. [38]. The antibiotics are as follows: amoxicillin (30 *μ*g/mL), ampilin (10 *μ*g/mL), sulphamethoxazole (25 *μ*g/mL), vancomycin (30 *μ*g/mL), cefpodoxime (10 *μ*g/mL), chloramphenicol (30 *μ*g/mL), oxacilin (1 *μ*g/mL), entrapenin (10 *μ*g/mL), azithromycin (30 *μ*g/mL), gentamicin (10 *μ*g/mL), ciprofloxacin (30 *μ*g/mL), erythromycin (16 *μ*g/mL), tetracycline (30 *μ*g/mL), and nitrofurantoin (300 *μ*g/mL). About 0.2 mL of the bacterial suspension (equivalent to the McFarland standard value of 0.5 containing 2.5 × 10^7^ CFU/mL) was spread onto Muller–Hinton agar (MHA) plates using a sterile cotton swab. The antibiotic discs were placed on the surface of the agar plates, which were maintained at 400°C for 2 h for diffusion. The diameter (millimeter) of the inhibition zone was measured after incubation at 370°C for 48 h.

The antibacterial activity of culture-free supernatant (CFS) from the LAB strain against some test pathogens (*Escherichia coli*, *Salmonella typhimurium*, *Aeromonas hydrophila*, *Klebsiella pneumonia*, *Bacillus subtilis*, *Staphylococcus aureus*, and *Proteus* sp.) was done using the agar-well diffusion method [38]. The sterile nutrient agar was seeded with 0.2 mL of the tested pathogens (containing 1.5 × 10^7^ CFU/mL), allowed to solidify, and 6 mm wells were bored on the agar with a sterile cork borer. About 40 *μ*L of filtered sterilized CFS was added to each well and allowed to diffuse for 1 h, followed by incubation at 370°C for 24 h. The diameter of the zone of inhibition around each well was measured. The experiment was performed in triplicate.

#### 2.5.1. Safety Evaluation of Isolate

#### 2.5.2. Determination of Haemolysis, Deoxyribonuclease (DNase), and Lecithinase Activity of the Isolate

The haemolytic activity of the isolate was evaluated using a method adapted from Yadav, Puniya, and Shukla [39]. The isolates were plated onto blood agar plates containing 5% sheep blood and incubated at 37°C for 48 h. Following incubation, the plates were examined for beta, alpha, and nonhaemolytic activities.

The isolate was cultured on DNase agar medium to assess its ability to produce the DNase enzyme. The plates were incubated at 37°C for 48 h and then examined for a clear pinkish zone around the colonies, which indicated positive DNase activity, as described by Shuhadha et al. [40].

The isolate was cultured on lecithinase agar medium to evaluate their ability to break down lecithin. The plates were incubated at 37°C for 48 h and then examined for a clear white opaque zone of precipitation that extended beyond the edge of the colony, indicating positive lecithin activity, as described by Cappuccino and Sherman [41].

### 2.6. Application of the EPS Produced From the LAB Strain for the Production of Probiotic Fermented Coconut Beverage

#### 2.6.1. Substrate Preparation, Formulation, and Production of Enriched Coconut Yogurt

The EPS produced was used in the formulation of coconut yogurt samples. The five coconut kernels were shelled and peeled to remove the outer brown skin and washed with portable water to remove all the dirt. One kilogram of the washed coconut was chopped into pieces before grating into fine particles (0.2 mm) [42, 43].

Substrates such as milk, grated coconut, and 3% (1.5 g) of starter culture, EPS, and 1.5% of LAB strain were added in varied concentrations for the formulation of probiotic fermented coconut beverage. Twelve formulations containing varied substrate concentrations labeled A1, A2, A3, and A4; B1, B2, B3, and B4; and CI, C2, C3, and C4, respectively, as shown in Table 1, were used (substrate formulation).

The formulated samples were pasteurized at 90°C for 30 min; the samples and the control samples were allowed to ferment at 44°C for 6 h and stored at 4°C over a period of 14 days. The samples were kept for further analysis.

### 2.7. Physicochemical Characterization of the Formulated Probiotic Coconut Yogurt Samples

The fermented probiotic samples were analyzed for pH, lactic acid (TTA), color, total soluble solid (TSS), specific gravity, and Vitamin C.

Determination of Lactic acid, pH, Colour, Total Soluble Solids and Vitamin C content of the stored Formulated Probiotic Coconut Yogurt Samples.

The lactic acid content of the stored, formulated probiotic fermented coconut beverage samples was determined by titrating 10 mL of the homogenized sample against 0.25 mol^−1^ NaOH using phenolphthalein as an indicator. Each milliliter of 1 N NaOH is equivalent to 90.08 mg of lactic acid [44].

The color of the stored formulated probiotic coconut yogurt samples was determined using a colorimeter. The color parameters (Hunter L∗, lightness; *a*∗, redness; and *b*∗, yellowness) were determined for each sample using a colorimeter (CM-2500D Minolta, Japan) [45].

TSSs of the stored formulated probiotic fermented coconut beverage samples were evaluated using a hand refractometer (Erma, Japan) in terms of °Bx (°Brix) [46].

The Vitamin C content of the stored formulated probiotic fermented coconut beverage samples was determined by titrating 10 mL of filtered samples against 0.01% blue 2,6-dichloro-phenolindophenol solution [40]. The final point was considered when the solution reached a pink color for 30 s. The calibration curve was performed with a Vitamin C solution, and the results were expressed as milligrams/100 mL ascorbic acid.

### 2.8. Determination of the Proximate Mineral Composition of the Stored, Formulated Probiotic Fermented Coconut Beverage Samples

The proximate composition (moisture, fat, crude fiber, crude protein, ash, and carbohydrate contents) of the stored formulated probiotic fermented coconut beverage samples was determined [47]. The determination was done in duplicate.

The mineral composition (Mg, K, and Ca content) of the stored formulated probiotic fermented coconut beverage samples was determined using the absorption spectrometer method of AOAC [47] and Kirk and Sawyer [48].

### 2.9. Determination of the Shelf-Life Extension of the Formulated Coconut Yogurt

The shelf-life extension of the stored formulated fermented coconut beverage samples was done by pour plating them at intervals of 7 days [49]. Serially diluted samples were pour-plated on nutrient agar for total heterotrophic count, MacConkey agar for total coliform count, and MRS for LAB count. The plates were incubated at 37°C for 48 h; distinct colonies were counted and reported as colony-forming units, while hazy colonies were designated as too numerous to count (TNTC).

### 2.10. Molecular Characterization

The selected probiotic strains were identified using 16S rRNA [50]. The genomic DNA was extracted using ZR Bacterial DNA MiniPrep (manufactured by Zymo Research). The DNA was PCR amplified using 27F-1492R primers under standard conditions. PCR amplification was done using an automated thermal cycler (Biometra, Germany) using Taq polymerase (TaKaRa Bio, Inc., Shiga, Japan). The amplified products were sequenced and analyzed using BLAST (NCBI, Bethesda, MD, United States). The neighbor-joining phylogenetic tree was constructed using MEGA 5.05.

### 2.11. Statistical Analysis

All experiments were carried out in triplicate, and each sample was analyzed in duplicate. The results are expressed as mean ± S.D. (standard deviation).

## 3. Results and Discussion

### 3.1. Production and Characterization of EPS Produced by Isolate W3

**Figure 1:**
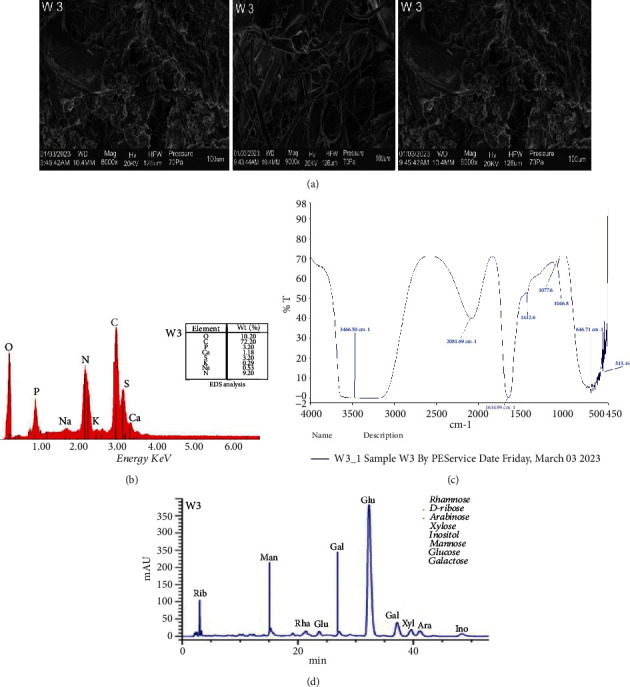
(a) Scanning electron micrograph of the EPS produced by Isolate W3. (b) Energy-dispersive X-ray spectra of W3 EPS sample. (c) Fourier transform infrared (FT-IR) spectra of W3 EPS samples. (d) High-performance liquid chromatogram (HPLC) of W3 EPS samples.

The EPS produced by the isolate was 6204.50 mg/L. The scanning electron micrograph of the EPS produced by the LAB strain is shown in Figure 1(a). The EPS was a highly compacted flake-like structure. Yadav et al. [51] had similar report on novel EPS from *Lactobacillus fermentum*. SEM analysis provides detailed insights into the morphological structure of the EPS.

Figure 1(b) shows the chemical composition of the EPS, which was determined by energy-dispersive X-ray spectroscopy. The elemental analysis revealed the predominance of oxygen and carbon with a mass ratio of 10.20–72.20 (*w*/*w*) for the EPS. The high mass of these two elements emphasized that the purified EPS is comprised mainly of carbohydrates [52]. The EDX spectrum also revealed the presence of other elements such as P, Ca, S, K, Na, and N. The elemental composition of the EPS was similar to the EPS produced by *Rhodobacter johrii* CDR-SL 7Cii [52]. Such elements present on the EPS surface may play a role in binding with the carboxyl and hydroxyl groups of monosaccharides [53]. EPSs that contain carbon, oxygen, and nitrogen are called glycoproteins, as they are composed of polysaccharides covalently linked to proteins.

The FT-IR spectra of the LAB EPS are shown in Figure 1(c). Eight peaks were observed, starting from 3466.50 to 515.46 cm^1^. The peaks at 3466.50, 2081.69, and 1634.99 cm^1^ could be attributed to the hydroxy group, H-bonded OH stretch, transition metal carbonyls, and the presence of Amide II band. The peak at 1412.60, 1077.60, and 1046.80 cm^1^ corresponds to vinyl C-H in-plane bend, primary amine (C-N) stretch, and P III polysaccharide band C-O-C group. The peaks at 646.71 and 515.46 cm^1^ correspond to aliphatic bromo compounds (C-Br) stretching. The IR spectrum revealed characteristic signatures and distinctive functional groups of carbohydrates. The assignments of the IR bands were mainly based on the previously reported polysaccharide spectra [54, 55].

The HPLC chromatogram of the EPS is shown in Figure 1(d).  Table 2 shows the monosaccharide composition of the EPS produced by the isolate. EPS contains eight monosaccharaides, which include rhamnose, D-ribose, arabinose, xylose, inositol, mannose, glucose, and galactose in a varied molar ratio. The monosaccharides had varying concentrations, with glucose having the highest and inositol having the lowest. EPSs produced by LAB are composed of various monosaccharides, including glucose, galactose, rhamnose, mannose, and N-acetylgalactosamine [54–56]. The specific monosaccharide composition can vary depending on the LAB strain. For example, EPS from *Streptococcus thermophilus* strains typically contains glucose and galactose [56]. *S. thermophilus* CC30 EPS is composed of glucose and galactose, while *S. thermophilus* LY03 EPS contains glucose, galactose, and N-acetylgalactosamine [56]. The occurrence of different saccharide moieties suggests that the EPS produced is a heteropolysaccharide.

**Figure 2:**
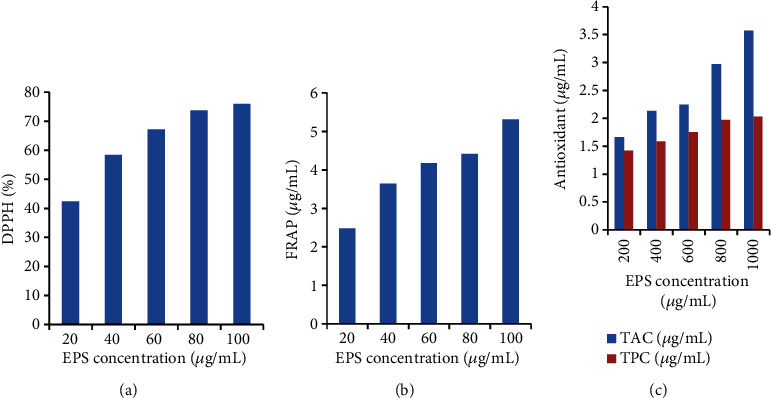
Antioxidant: (a) DPPH; (b) FRAP; (c) TAC and TPC potential of the EPS sample produced by Isolate W3.

Figures 2(a), 2(b), and 2(c) show the in vitro antioxidant activity of the EPS in terms of DPPH radical scavenging activity, ferric ion-reducing property, total antioxidant capacity, and the TP content of the EPS. The DPPH and FRAP ranged from 42.36% to 75.88% and 2.48 to 5.31 *μ*g/mL, respectively. The DPPH has IC_50_ of 27.299. The TAC and TPC ranged from 1.66 to 3.57 *μ*g/mL and 1.42 to 2.03 *μ*g/mL, respectively, in which the highest activity was recorded at highest concentration. The antioxidant activity increased in a dose-dependent manner. As the concentration increased, the antioxidant potential increased. The in vitro antioxidant assay showed that EPS has strong antioxidant properties. EPSs produced by LAB have been studied for their antioxidant properties, which can be assessed through various in vitro methods. These methods include evaluating the EPS's ability to scavenge DPPH radicals, reduce ferric ions, exhibit total antioxidant capacity, and contain TP compounds. Ability of the EPS to effectively neutralized free radicals as evidenced by its significant DPPH radical scavenging activity, with values ranging from 42.36% to 75.88%, indicates the potential of EPS to combat oxidative stress. Studies have shown that EPS extracted from spent media wastewater can effectively neutralize free radicals, 71.6%–79.1% [57]. The FRAP values of the EPS, ranging from 2.48 to 5.31 *μ*g/mL, demonstrate its capacity to act as an electron donor and react with free radicals, thereby terminating free radical chain reactions. This property is crucial for protecting cells from oxidative damage. The total antioxidant capacity and TPC of the EPS, with concentrations ranging from 1.66 to 3.57 *μ*g/mL and 1.42 to 2.03 *μ*g/mL, further highlight its substantial antioxidant potential. The presence of phenolic compounds in EPS can contribute to its antioxidant properties. Phenolic compounds are known for their ability to scavenge free radicals and exhibit antioxidant effects. EPSs derived from various sources, such as *Lactobacillus plantarum* C88 and *Lysobacter soyae* sp. nov, have shown promising antioxidant activities, making them valuable candidates for applications in functional foods and other industries [57, 58]. The antioxidant properties of EPS can vary based on their monosaccharide composition and structural characteristics, emphasizing their potential as natural antioxidants with high thermal stability and dose-dependent antioxidant effects.

#### 3.1.1. Probiotic Evaluation of the LAB Strain

**Figure 3:**
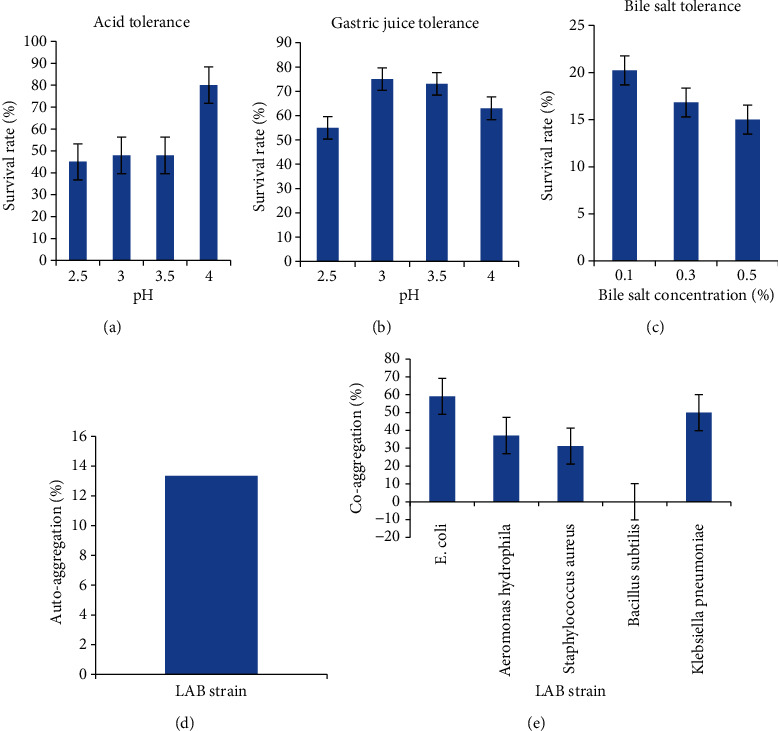
Tolerance of the Isolate W3 to (a) acid, (b) gastric juice, and (c) bile salt. LAB strain: (d) autoaggregation and (e) coaggregation with some selected microorganisms.

The survival rate of the LAB strain to acid, gastric juice, and bile salt is shown in Figures 3(a), 3(b), and 3(c), respectively. There was a significant difference (*p* < 0.05) in the survival rate of the isolate at different acid levels. The survival rate of the isolate at different acid concentrations ranged from 45% - 80%, with the highest survivability recorded at pH 4.0.

There was a significant difference in the survival rate of the isolate to gastric juice. The survival rate to gastric acid ranged from 55% to 75% OD. The highest survivability was recorded at pH 3.5 for gastric acid. One of the major stress factors facing ingested probiotics is their passage through the stomach and being subjected to gastric acids with a low pH value [59].

Probiotic microorganisms should be tolerant of gastric pH 3 [60, 61]. A study conducted by Zielińska et al. [62] reported a 30%–100% survival rate of probiotic *Lactobacillus* strains when inoculated in gastric juice at pH 3. The viability of the isolate at pH 3 and acidic gastric juice was found to be similar to other reported probiotic strains that are commonly reported in traditional food products [63, 64].

The isolate was able to survive at different concentrations of bile salt. The survival rate ranged from 14.99% to 20.25%, with the highest recorded at 0.1% bile salt concentration. The isolate had low survivability to high bile salt concentrations which may be due to the presence of a low specific enzyme called bile salt hydrolase (BSH), which helps to hydrolyze conjugated bile salts and minimize their toxicity [65].

The survival of LAB in stomach acidic conditions, gastric juice, and bile salts is essential for their potential as probiotics. LAB strains exhibit diverse levels of tolerance to these environments. For instance, *Lactobacillus plantarum* TDM41 and other isolates have shown survival rates exceeding 85% in acidic pH settings [66]. Some *Pediococcus acidilactici* isolates can endure gastric juice at pH 3 with a survival rate of 68%. LAB strains have been evaluated for their survival in bile salts, with selected isolates demonstrating resistance to 0.3% bile salt concentration [67, 68]. While a specific minimum survival rate for LAB to qualify as probiotics is not explicitly defined, a survival rate of at least 50% in acidic conditions, gastric juice, and bile salts is generally considered favorable. The minimum survival threshold may vary based on the intended application and desired probiotic effects [67].

Figure 3(d) shows the autoaggregation of Isolate W3 after 5 h. The autoaggregation increased with an average of 13.34 ± 0.01%, which signifies a lower ability to colonize and attach to the intestinal epithelium [58], which corroborates previous studies that reported that the LAB strains isolated from raw milk showed no or low autoaggregation [59, 60]. LAB strains with a reduced capacity to colonize and adhere to epithelial cells are considered probiotic because their efficacy as probiotics is not solely reliant on these abilities [69, 70]. Probiotic strains can deliver health benefits through various mechanisms beyond colonization, such as modulating the immune response, generating antimicrobial substances, and influencing the gut microbiota composition. Even if a LAB strain exhibits limited colonization and attachment capabilities, it can still provide positive effects in the gut by interacting with the host's immune system and fostering a healthy gut environment. Hence, the probiotic potential of LAB strains should be assessed based on a range of factors beyond their ability to colonize and adhere to epithelial cells [69, 70].

Autoaggregation experiments help in studying the colonization and adhesion of probiotic bacteria to epithelial cells in the gastrointestinal tract, which leads to the prevention of colonization by pathogens through their interaction [69].

Figure 3(e) shows the coaggregation of Isolate W3 with some test pathogens. The coaggregation ranged from 37.14% to 59.09%. Isolate W3 had the highest coaggregation with *E. coli* (59.09%) but was unable to coaggregate with *B. subtilis*. According to Solieri et al. [71], coaggregation values lower than 20% correspond to strains that have weak coaggregation ability, and based on this criterion, Isolate W3 possesses high coaggregating ability. This ability of strains prevents pathogens from colonizing the GI tract [72] and could help constitute an important defense mechanism against infection [73].

Table 3 shows the susceptibility of the probiotic isolate to some antibiotics. The susceptibility of the isolate ranged from 10.0 to 26 mm. It had the highest susceptibility to chloramphenicol (26.0 mm) and the lowest susceptibility to gentamicin (10.0 mm). The isolate was resistant to amoxicillin, ampilin, sulphamethoxazole, vancomycin, cefpodoxime, oxacilin, entrapenin, and azithromycin. The antibiotic resistance may be attributed to the cell wall structure, membrane permeability, and efflux mechanisms. Susceptibility to chloramphenicol, gentamicin, ciprofloxacin, erythromycin, tetracycline, and nitrofurantoin is in accordance with the work of Sukumar and Ghosh [74].

**Figure 4:**
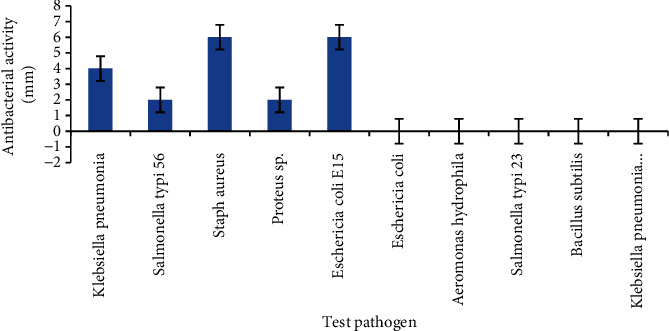
Antibacterial potential of the CFS or metabolites of Isolate W3 against some test pathogens.

Figure 4 shows the antibacterial potential of the CFS or metabolites of Isolate W3 against some test pathogens. The antibacterial potential of the metabolite of the Probiotic Strain W3 ranged from 2.0 to 8.0 mm. About 50% of the pathogenic bacteria were susceptible to the Metabolite W3. The Probiotic Strain W3 had the highest zone of inhibition against *Staphylococcus aureus* and *E. coli* (6.0 mm), and the lowest zone of inhibition was observed against *Salmonella typhi* and *Proteus* (2.0 mm). The result showed that Isolate W3 had potent antimicrobial activity with different inhibitory potentials. This antagonistic activity can be attributed to the antimicrobial substances produced by this LAB strain, which include organic acids (e.g., lactic acid and acetic acid), fatty acids, hydrogen peroxide, acetoin, diacetyl, and most importantly, inhibitory peptides known as bacteriocins [75]. Lack of antagonistic activity against some of the test isolates may be due to low concentration of the antimicrobial compounds in the CFS. The isolates are resistance to 8 out of the14 antibiotics tested. Probiotic LAB can exhibit resistance to antibiotics through various mechanisms, including efflux pumps and enzymatic inactivation. LAB can possess multidrug-resistant (MDR) efflux pumps that actively expel antibiotics from the bacterial cells, conferring resistance. Some LAB strains can produce enzymes that directly inactivate certain antibiotics, such as beta-lactams, rendering them ineffective.

The safety assessment of Isolate W3 for hemolysis, DNase, and lecithinase activity is shown in Table 4. The isolate was negative to all the hemolysis, DNase, and lecithinase negative. This indicates the safety of this isolate as a probiotic candidate. LABs that are negative to hemolysis, DNase, and lecithinase are considered safer for use in various applications, including food production and human health. The absence of these enzymes suggests a lower risk of harm to humans and the environment. These enzymes are associated with virulence factors that can contribute to the pathogenicity of bacteria, and their absence reduces the potential for adverse reactions in humans.

**Figure 5:**
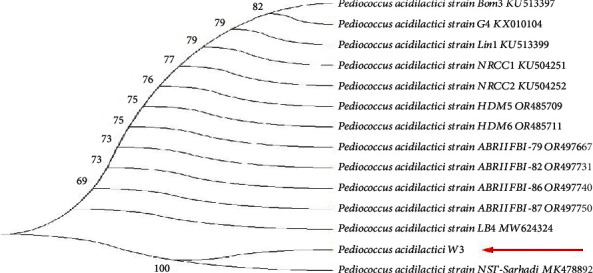
Phylogenetic tree of *Pediococcus acidilactici*.

Figure 5 shows the molecular identification and phylogenetic tree of the isolated genomic DNA amplified by PCR when analyzed using agarose gel electrophoresis. The isolate was identified to have a molecular weight of 1.5 k base pairs when compared with the standard markers. The phylogenetic tree was determined by comparing the homology with the existing GenBank database, and the comparative analysis revealed their closest neighbors. Isolate W3 was identified to have 100% pairwise identity to *Pediococcus acidilactici* strain NST-Sarhadi with NCBI accession number MK478892.1.

### 3.2. Physicochemical Property of the Stored Probioticated Fermented Coconut Beverage Samples Produced by the Isolate

Table 5(a) shows the physicochemical properties, such as pH, specific gravity, TSSs, lactic acid, and Vitamin C content, of the stored formulated probiotic yogurt sample.

There was a significant increase in pH during storage. On Days 0 and 7, the pH ranged from 5.31 to 5.53 and 6.1 to 6.8. Samples A2 and B1 had the highest pH on Days 0 and 7, respectively. It was observed that the pH of the formulated fermented coconut beverage samples increased during storage, which is in contrast to the work of Eke et al. [76, 77] and the food standard code that requires that the pH of yoghurt be a maximum of 4.50 to prevent the growth of any pathogenic microorganisms [78]. The increase in pH may be attributed to the metabolic activity of the LAB in the fermented medium which resulted in a reduction in acidity of the medium with a concomitant increase in pH. During LAB fermentation, pH can increase due to various factors. LAB consumes sugars, converting them into lactic acid, initially lowering pH. However, as sugar depletion slows lactic acid production, other metabolites like acetic acid and ethanol can mildly decrease pH. Some LAB strains can metabolize lactic acid, leading to slower pH decline or even an increase. Growth phase, substrate buffering capacity, and environmental factors such as temperature and oxygen availability also affect pH. Also, pH changes in LAB fermentation result from a complex interplay of metabolic processes, substrate composition, and environmental conditions. Understanding these factors is crucial for fermentation optimization.

Specific gravity is an important parameter to monitor during yogurt fermentation as it provides valuable information about the progress and completion of the process. This specific gravity range is essential for achieving the desired texture, body, and mouth feel of the final yogurt product. The normal value of specific gravity of coconut yoghurt typically ranges from 1.017 to 1.034. The specific gravity of the fermented probiotic coconut beverage samples ranged from 0.763 to 1.2743 kg/m^3^ and 0.910 to 1.573 kg/m^3^ at Days 0 and 7. There were significant changes in all the yogurt samples during storage. There was an increase in the specific gravity of some samples during storage for 7 days. There was a reduction in the specific gravity of Samples A2, A4, and B4 during storage for 7 days. The significant increase in the specific gravity (*p* ≤ 0.05) after storage could be attributed to increased total solids (soluble and insoluble). This is in agreement with the work of Imele and Atemnkeng [20], who reported an increase in fat content, specific gravity, and total solids with the addition of coconut milk to plain yoghurt. The specific gravity is mainly due to the presence of water contents and small concentrations of fats, proteins, vitamins, enzymes, and minerals in the sample [79].

The TSSs ranged from 0.099 to 0.194 °Bx and 0.104 to 0.196 °Bx at Days 0 and 7, respectively. Sample C2 had the highest TSS at Days 0 and 7 of storage. There was variation in the TSS during storage in which an increase in TTS was observed in all the samples except Samples B4 and C3. There was a reduction in TSS in some samples. The decrease can be related to the probiotic microorganisms that use up the sugars for fermentation. The increase may be due to the contribution of monosaccharide sugars from the addition of coconut [80, 81]. Falade et al. [80] reported a similar trend of increase in the TSSs for soy and Bambara plain yoghurt stored at 7°C. In contrast, Rasdhari et al. [81] reported that the TSSs of some probiotic yogurt products decreased during storage at 4°C for 7 days.

The lactic acid content of the stored formulated probioticated fermented coconut beverage samples ranged from 10.8096 to 27.9248 mg/L and 12.6112 to 55.8496 mg/L after Day 7. There was a significant difference in lactic acid content in all the formulated yogurt samples. Sample A2 with commercial starter had the highest lactic acid content at Day 7 of storage, while Sample C1 enriched with 0.5% EPS had the lowest lactic acid content at Day 7 of storage.

The increase in lactic acid content of the yogurt products during the storage period could be attributed to the starter culture's activity, such as postacidification due to the formation of lactic acid or the growth of the bacteria used during fermentation [82, 83]. Falade et al. [80] reported a similar trend in the total titratable acidity of plain soy and Bambara yoghurts stored at 7°C for 9 days.

The increase in pH and lactic acid content occurring simultaneously could be attributed to the metabolic activity of the probiotic and the commercial LAB starter during substrate fermentation. These bacteria convert lactose into lactic acid, leading to a decrease in pH, but variations in the fermentation process, such as the specific strains of LAB used, incubation conditions, and the presence of EPS, could potentially result in a temporary increase in pH alongside lactic acid production [84, 85]. The increase can equally be attributed to longer fermentation time.

The Vitamin C content ranged from 0.40% to 0.92% and 0.31% to 0.66% at Days 0 and 7. There was a significant difference (*p* < 0.05) in the Vitamin C content in all the formulated samples across the days. Samples B4 and A4 with the highest concentration of coconut had the highest Vitamin C content at Days 0 and 7, while Sample B1 without coconut milk had the lowest Vitamin C content at Day 7. Reduction in Vitamin C could be attributed to the heating process involved in yogurt making, which can lead to the degradation of Vitamin C content in the milk used for fermentation, as well as the utilization of Vitamin C by the bacterial culture used in the production of yogurt.

Table 5(b) shows the proximate composition of the stored formulated probiotic fermented coconut beverage samples at Days 0 and 7. The ash content, crude fiber content, and fat content ranged from 0.55% to 1.6% (Day 0) and 0.4% to 1.35% (Day 7), 0.125% to 0.68% (Day 0) and 0.15% to 0.45% (Day 7), 0.1% to 0.4% (Day 0), and 0.1% to 0.35% (Day 7), respectively. The moisture content, crude protein content, and carbohydrate content ranged from 30.6% to 89.8% (Day 0) and 61.1% to 94.9% (Day 7), 0.08% to 5.61% (Day 0), 0.23% to 4.26% (Day 7), 7.465% to 65.00% (Day 0), and 0.785% to 35.615% (Day 7). Sample C1 had the highest fat and protein content during storage.

An increase in ash content is often linked to the metabolic processes of the starter cultures and probiotic LAB, resulting in higher mineral levels, while a reduction in ash content, though less common, could occur if the balance between starter cultures and milk components is suboptimal, potentially limiting LAB activity. This agreed with the variation in the formulation of the samples. The absence of significant changes in ash content during yogurt fermentation may stem from the minimal impact of the starter cultures and LAB on the milk's mineral composition [86–88].

A decrease in crude fiber and moisture content during coconut yogurt fermentation using commercial starter and probiotic LAB with EPS is likely due to the metabolic activity of the cultures, which may break down some of the fiber components and utilize moisture for growth. An increase in crude fat and carbohydrate content could be attributed to the addition of ingredients like EPS, which can elevate the fat and carbohydrate levels in the final yogurt product. The production of EPS by the probiotic LAB may also contribute to an increase in carbohydrate content. Reduction in fat content may be attributed to the lipolytic activity of the starter during fermentation.

A reduction in protein could be due to the breakdown of proteins by LAB, while an increase in protein content could result from the utilization of protein-rich ingredients like coconut milk in the fermentation process. The specific changes in protein content are influenced by the metabolic activity of the cultures, the composition of the milk used, and the presence of additional protein sources in the yogurt mixture [89].

Table 5(c) shows the mineral composition of the stored, formulated probiotic yogurt samples at Days 0 and 7. The Ca, K, and Mg content ranged from 43.678–87.863 mg/100 g (Day 0) to 55.832–162.302 mg/100 g (Day 7), 12.079–15.121 mg/100 g (Day 0), and 10.259–13.536 mg/100 g (Day 7) to 32.04–70.32 mg/100 g (Day 0) and 36.11–61.12 mg/100 g (Day 7). Sample B1 had the highest Ca and Mg content at Day 0, while Sample B4 had the highest K content at Day 0. On Day 7, Sample C4 had the highest Ca and K content, while Sample A4 had the highest Mg content.

An increase in calcium, magnesium, and potassium content during fermented probiotic coconut beverage production may be attributed to some factors such as the following: an addition of mineral-rich ingredients such as coconut milk, which is a good source of minerals, which can increase the calcium, magnesium, and potassium content in the final yogurt product; metabolic activity of probiotic LAB for the production process which may release minerals like potassium into the yogurt mixture; and the fermentation conditions such as temperature and duration which may affect the availability and solubility of minerals in the yogurt mixture [90]. Reduction in Ca, Mg, and K may be attributed to the anabolic activity of the isolates during fermentation process, which make use of the minerals for biosynthetic activities such as growth and catalytic processes [91].

Table 6 shows the viability/survivability of the probiotic strain in the stored yoghurt samples which indicates a decrease in the viability after 7 days of storage compared to Day 0 except for Samples A2 and A3. The increase observed in Samples A2 and A3 could be a result of the interaction between the probiotic strain and some components in the yoghurt resulting in an increase in metabolic activity and promoting its viability over time. No observable growth was recorded in Samples A2, A4, and B2. This could be a result of nutrient exhaustion which inhibits metabolic activity resulting in loss of viability of probiotic strain over time.

Higher growth rate recorded in Sample A2 at Day 7 with a lower initial load at Day 0 may be as a result of less competition for nutrients and resources, allowing the starter to proliferate more rapidly. Conversely, in the Sample A4 with a higher initial load at Day 0, competition for nutrient may inhibit the growth of LAB, resulting in slower growth rates. In Sample A4, the isolate might have used up the available substrate which resulted into nutrients in availability and reduction in growth.

## 4. Conclusion

The LAB isolated from *Wara* was an EPS producer. The EPS produced by the isolate was a flake-like heteropolymeric substance. The LAB metabolites had antibacterial activity against some of the test pathogens. FT-IR spectrum analysis revealed characteristic signatures and distinctive functional groups of carbohydrates in the EPS. HPLC analysis identified eight monosaccharides in the EPS, indicating a heteropolysaccharide composition. In vitro antioxidant assays demonstrated strong antioxidant properties of the EPS, as evidenced by DPPH radical scavenging activity, ferric ion-reducing property, total antioxidant capacity, and TP content. The LAB strain exhibited some level of significant tolerance to acid, gastric juice, and bile salt, indicating its potential to survive the gastrointestinal tract environment and, hence, has good probiotic properties. Autoaggregation and coaggregation assays showed moderate autoaggregation and high coaggregation abilities, respectively, which could aid in pathogen exclusion and colonization. Antibiotic susceptibility testing revealed varying degrees of susceptibility to different antibiotics, along with potent antibacterial activity against some of the pathogens tested. The LAB strain has 100% relatedness to *Pediococcus acidilactici* and exhibited a good starter for the production of fermented probiotic coconut beverage enriched with EPS. The probioticated fermented coconut beverage samples had significant pH development, varying lactic acid production, specific gravity, TSSs, Vitamin C content, proximate, and mineral composition during storage. Proximate analysis revealed variations in ash, crude fiber, fat, moisture, protein, and carbohydrate content, which could be influenced by the composition of the yogurt samples and storage conditions. Mineral composition analysis indicated fluctuations in calcium, magnesium, and potassium content during storage, highlighting the dynamic nature of these fermented probiotic coconut beverage samples. The fermented probiotic coconut beverage samples have good nutritional characteristics, which could serve as a functional and natural nutraceutical food for lactose-intolerant populations and dairy-allergic individuals.

## Figures and Tables

**Table 1:** Substrate formulation.

**Ingredients**	**Sample code**											
	**A1**	**A2**	**A3**	**A4**	**B1**	**B2**	**B3**	**B4**	**C1**	**C2**	**C3**	**C4**
Milk (%)	70	70	70	70	100	90	80	70	70	70	70	70
Coconut (%)	30	30	30	30	0	10	20	30	30	30	30	30
Starter culture (%)	0	1.5	0	0	1.5	1.5	1.5	1.5	1.5	1.5	1.5	1.5
Exopolysaccharide (%)	0	0	1.5	0	0	0	0	0	0.5	1.0	1.5	2.0
LAB (%)	1.5	0	0	0	1.5	1.5	1.5	1.5	1.5	1.5	1.5	1.5

**Table 2:** The monosaccharide composition of the EPS produced by Isolate W3.

**S/N**	**Retention time**	**Peak area (%)**	**Name of compound**	**Molecular weight**
1	4.09	100	Ribose	80
2	15.3	200	Mannose	160
3	22.5	10	Rhamnose	88
4	23.1	10	Glucose	95
5	27.2	240	Galactose	150
6	32.6	370	Glucose	164
7	37.9	50	Galactose	132
8	38.7	30	Xylose	166
9	40.1	20	Arabinose	220
10	44.2	15	Inositol	150

**Table 3:** Antibiotic susceptibility of Isolate W3 to some selected antibiotics.

**Antibiotics (*μ*g)**	**Zone of inhibition (mm)**
Amoxicillin (30)	0.00
Ampilin (10)	0.00
Sulphamethoxazole (25)	0.00
Vancomycin (30)	0.00
Cefpodoxime (10)	0.00
Chloramphenicol (30)	26.0
Oxacilin (1)	0.00
Entrapenin (10)	0.00
Azithromycin(30)	0.00
Gentamicin (10)	10.0
Ciprofloxacin (30)	12.0
Erythromycin (16)	24.0
Tetracycline (30)	14.0
Nitrofurantoin (300)	20.0

**Table 4:** Safety assessment of Isolate W3.

	**Safety evaluation**
Safety test	Isolate W3
Hemolysis	—
DNase	—
Lecithinase	—

**(a):** 

**Sample code**	**pH**		**Specific gravity (kg/m** ^**3**^ **)**		**Total soluble solid (Brix)**		**Lactic acid content (mg/L)**		**Vitamin C content (%)**	
	**Day 0**	**Day 7**	**Day 0**	**Day 7**	**Day 0**	**Day 7**	**Day 0**	**Day 7**	**Day 0**	**Day 7**
A1	5.48 ± 0.01^b^	6.05 ± 0.07^a^	0.975 ± 0.00^b^	1.157 ± 0.00^a^	0.135 ± 0.00^a^	0.136 ± 0.00^a^	17.13 ± 0.01^b^	30.52 ± 0.02^a^	0.76 ± 0.01^a^	0.53 ± 0.00^b^
A2	5.52 ± 0.02^b^	6.40 ± 0.14^a^	1.127 ± 0.00^b^	1.143 ± 0.00^a^	0.131 ± 0.00^b^	0.137 ± 0.00^a^	15.32 ± 0.01^b^	55.87 ± 0.02^a^	0.78 ± 0.02^a^	0.61 ± 0.01^b^
A3	5.40 ± 0.01^b^	6.45 ± 0.21^a^	1.245 ± 0.00^b^	1.414 ± 0.00^a^	0.135 ± 0.00^b^	0.141 ± 0.00^a^	18.91 ± 0.01^b^	32.44 ± 0.01^a^	0.62 ± 0.01^a^	0.54 ± 0.01^b^
A4	5.48 ± 0.01^b^	6.40 ± 0.00^a^	1.247 ± 0.00^a^	1.136 ± 0.00^b^	0.147 ± 0.00^b^	0.149 ± 0.00^a^	10.80 ± 0.01^b^	41.44 ± 0.01^a^	0.88 ± 0.00^a^	0.65 ± 0.01^b^
B1	5.38 ± 0.01^b^	6.85 ± 0.07^a^	1.275 ± 0.00^b^	1.573 ± 0.01^a^	0.173 ± 0.00^a^	0.176 ± 0.00^a^	18.01 ± 0.01^b^	3.60 ± 0.01^a^	0.53 ± 0.00^a^	0.26 ± 0.00^b^
B2	5.36 ± 0.00^b^	6.65 ± 0.07^a^	0.763 ± 0.00^b^	0.910 ± 0.00^a^	0.101 ± 0.00^a^	0.104 ± 0.00^a^	19.83 ± 0.01^a^	13.49 ± 0.03^b^	0.45 ± 0.01^a^	0.30 ± 0.01^b^
B3	5.35 ± 0.01^b^	6.50 ± 0.00^a^	0.930 ± 0.00^b^	1.129 ± 0.00^a^	0.176 ± 0.00^a^	0.179 ± 0.00^a^	24.33 ± 0.01^a^	20.71 ± 0.01^b^	0.75 ± 0.01^a^	0.44 ± 0.00^b^
B4	5.46 ± 0.00^b^	6.10 ± 0.14^a^	1.262 ± 0.00^a^	1.065 ± 0.01^b^	0.172 ± 0.00^a^	0.168 ± 0.00^a^	21.64 ± 0.02^b^	27.04 ± 0.03^a^	0.91 ± 0.01^a^	0.67 ± 0.01^b^
C1	5.41 ± 0.01^b^	6.50 ± 0.00^a^	1.128 ± 0.00^b^	1.215 ± 0.00^a^	0.175 ± 0.00^a^	0.177 ± 0.00^a^	18.03 ± 0.01^a^	12.62 ± 0.01^b^	0.44 ± 0.00^a^	0.32 ± 0.01^b^
C2	5.44 ± 0.00^b^	6.30 ± 0.00^a^	1.245 ± 0.00^b^	1.397 ± 0.00^a^	0.193 ± 0.00^a^	0.197 ± 0.00^a^	18.91 ± 0.02^b^	24.30 ± 0.03^a^	0.62 ± 0.01^a^	0.41 ± 0.01^b^
C3	5.32 ± 0.01^b^	6.55 ± 0.07^a^	1.192 ± 0.00^b^	1.286 ± 0.00^a^	0.177 ± 0.00^a^	0.137 ± 0.00^b^	27.94 ± 0.01^a^	13.51 ± 0.00^b^	0.41 ± 0.01^a^	0.39 ± 0.01^a^
C4	5.37 ± 0.01^b^	6.30 ± 0.00^a^	1.206 ± 0.00^b^	1.294 ± 0.00^a^	0.171 ± 0.00^a^	0.176 ± 0.00^a^	21.64 ± 0.02^b^	23.42 ± 0.00^a^	0.54 ± 0.01^a^	0.44 ± 0.01^b^

*Note:* Each value is a mean ± standard error of three replicates. Values in the same row with different letters are significantly different by Duncan multiple range test (*p* < 0.05).

Abbreviations: A: milk = 70%, coconut = 30%; A1: A + 1.5% LAB; A2: A + 1.5% C.S; A3: A + 1.5% EPS; A4: A; B1: 100%milk + 1.5%LAB + 1.5%C.S; B2: 90%milk + 10%coconut + 1.5%LAB + 1.5%C.S; B3: 80%milk + 20%coconut + 1.5%LAB + 1.5%C.S; B4: 70%milk + 30%coconut + 1.5%LAB + 1.5%C.S; C1: A + 1.5%LAB + 1.5%C.S + 0.5%EPS; C2: A + 1.5%LAB + 1.5%C.S + 1.0%EPS; C3: A + 1.5%LAB + 1.5%C.S + 1.5%EPS; C4: A + 1.5%LAB + 1.5%C.S + 2.0%EPS.

**(b):** 

**Sample code**	**Proximate composition (%)/parameter**											
	**Ash content**		**Crude fiber**		**Fat content**		**Moisture content**		**Crude protein**		**Carbohydrate**	
	**Day 0**	**Day 7**	**Day 0**	**Day 7**	**Day 0**	**Day 7**	**Day 0**	**Day 7**	**Day 0**	**Day 7**	**Day 0**	**Day 7**
A1	1.2^a^	1.05^b^	0.53^a^	0.43^a^	0.1^a^	0.1^a^	61.6^a^	61.1^a^	1.65^b^	1.71^a^	34.93^a^	35.62^a^
A2	1.25^a^	1.1^b^	0.35^a^	0.45^a^	0.25^a^	0.1^b^	67.1^b^	75.6^a^	1.56^a^	1.5^a^	29.49^a^	21.25^a^
A3	1.6^a^	1.3^b^	0.28^a^	0.28^a^	0.3^a^	0.25^a^	75.5^b^	88.5^a^	0.08^b^	0.23^a^	22.25^a^	9.46^b^
A4	0.65^a^	1.0^b^	0.25^a^	0.23^a^	0.1^a^	0.1^a^	81.8^a^	74.4^b^	1.18^a^	1.11^a^	16.02^a^	23.17^b^
B1	1.1^b^	1.35^a^	0.68^a^	0.38^b^	0.1^a^	0.1^a^	30.6^b^	94.9^a^	2.52^a^	2.49^a^	65.05^a^	0.79^b^
B2	0.9^a^	0.7^b^	0.55^a^	0.4^a^	0.1^a^	0.1^a^	53.6^a^	54.3^a^	3.88^b^	4.0^a^	40.97^a^	40.5^b^
B3	1.05^b^	1.2^a^	0.13^a^	0.13^a^	0.1^a^	0.1^a^	89.8^a^	90.3^a^	1.23^a^	1.25^a^	7.69^a^	7.03^b^
B4	1.45^a^	1.0^b^	0.23^a^	0.18^a^	0.15^a^	0.2^a^	78.4^b^	80.0^a^	5.29^a^	4.2^b^	14.49^a^	14.43^b^
C1	0.6^a^	0.4^b^	0.35^a^	0.28^a^	0.45^a^	0.35^b^	70.3^a^	77.7^a^	5.61^a^	4.26^b^	22.69^a^	17.02^b^
C2	0.55^a^	0.5^a^	0.33^a^	0.23^a^	0.35^a^	0.25^b^	75.2^a^	68.6^a^	4.91^a^	4.19^a^	18.67^a^	26.24^b^
C3	0.7^b^	0.9^a^	0.23^a^	0.2^a^	0.3^a^	0.25^a^	90.0^a^	91.7^a^	1.31^a^	1.32^a^	7.47^a^	5.63^b^
C4	0.65^a^	0.65^a^	0.13^a^	0.15^a^	0.3^a^	0.25^a^	68.8^b^	80.7^a^	3.57^a^	2.75^b^	26.56^a^	15.5^b^

*Note:* Values in the same row with different letters are significantly different by Duncan's multiple range test (*p* < 0.05).

Abbreviations: A: milk = 70%, coconut = 30%; A1: A + 1.5%LAB; A2: A + 1.5%C.S; A3: A + 1.5%EPS; A4: A; B1: 100%milk + 1.5%LAB + 1.5%C.S; B2: 90%milk + 10%coconut + 1.5%LAB + 1.5%C.S; B3: 80%milk + 20%coconut + 1.5%LAB + 1.5%C.S; B4: 70%milk + 30%coconut + 1.5%LAB + 1.5%C.S; C1: A + 1.5%LAB + 1.5%C.S + 0.5%EPS; C2: A + 1.5%LAB + 1.5%C.S + 1.0%EPS; C3: A + 1.5%LAB + 1.5%C.S + 1.5%EPS; C4: A + 1.5%LAB + 1.5%C.S + 2.0%EPS.

**(c):** 

**Sample code**	**Mineral composition (mg)/storage time (days)**					
	**Ca**		**Mg**		**K**	
	**0**	**7**	**0**	**7**	**0**	**7**
A1	62.51 ± 0.01^a^	55.84 ± 0.01^b^	14.39 ± 0.00^a^	11.52 ± 0.01^b^	41.12 ± 0.01^a^	36.11 ± 0.00^b^
A2	68.96 ± 0.01^b^	73.11 ± 0.01^a^	14.25 ± 0.01^a^	12.54 ± 0.01^b^	43.25 ± 0.00^b^	49.53 ± 0.00^a^
A3	73.65 ± 0.00^b^	81.13 ± 0.01^a^	14.23 ± 0.01^a^	13.23 ± 0.01^b^	49.07 ± 0.01^b^	56.04 ± 0.01^a^
A4	68.01 ± 0.00^b^	77.26 ± 0.01^a^	14.14 ± 0.00^a^	13.54 ± 0.00^b^	41.12 ± 0.01^b^	54.49 ± 0.01^a^
B1	87.86 ± 0.00^a^	67.40 ± 0.01^b^	15.14 ± 0.02^a^	10.77 ± 0.01^b^	63.59 ± 0.01^a^	43.11 ± 0.01^b^
B2	79.89 ± 0.02^a^	77.05 ± 0.00^b^	14.68 ± 0.00^a^	12.12 ± 0.01^b^	50.37 ± 0.01^b^	52.26 ± 0.03^a^
B3	65.11 ± 0.01^b^	91.80 ± 0.01^a^	14.60 ± 0.01^a^	12.82 ± 0.00^b^	47.10 ± 0.01^b^	62.49 ± 0.02^a^
B4	74.39 ± 0.02^b^	139.30 ± 0.00^a^	14.00 ± 0.01^a^	12.85 ± 0.01^b^	70.32 ± 0.00^a^	55.02 ± 0.01^b^
C1	61.66 ± 0.01^b^	84.46 ± 0.00^a^	13.79 ± 0.01^a^	12.06 ± 0.01^b^	33.43 ± 0.02^b^	57.63 ± 0.00^a^
C2	71.78 ± 0.02^b^	85.11 ± 0.01^a^	12.75 ± 0.02^a^	12.31 ± 0.01^b^	34.58 ± 0.02^b^	57.63 ± 0.00^a^
C3	43.68 ± 0.00^b^	86.38 ± 0.00^a^	12.10 ± 0.02^b^	12.38 ± 0.02^a^	32.05 ± 0.01^b^	55.09 ± 0.01^a^
C4	49.25 ± 0.01^b^	162.31 ± 0.01^a^	14.29 ± 0.01^a^	13.00 ± 0.01^b^	37.53 ± 0.01^a^	61.13 ± 0.01^a^

*Note:* Each value is a mean ± standard error of three replicates. Values in the same row with different letters are significantly different by Duncan's multiple range test (*p* < 0.05).

Abbreviations: A: milk = 70%, coconut = 30%; A1: A + 1.5%LAB; A2: A + 1.5%C.S; A3: A + 1.5%EPS; A4: A; B1: 100%milk + 1.5%LAB + 1.5%C.S; B2: 90%milk + 10%coconut + 1.5%LAB + 1.5%C.S; B3: 80%milk + 20%coconut + 1.5%LAB + 1.5%C.S; B4: 70%milk + 30%coconut + 1.5%LAB + 1.5%C.S; C1: A + 1.5%LAB + 1.5%C.S + 0.5%EPS; C2: A + 1.5%LAB + 1.5%C.S + 1.0%EPS; C3: A + 1.5%LAB + 1.5%C.S + 1.5%EPS; C4: A + 1.5%LAB + 1.5%C.S + 2.0%EPS.

**Table 6:** Viability of the isolate in the stored formulated probioticated yoghurt samples.

**Survivability (× 10** CFU**/mL)/storage time (days)**								
**Sample code**	**Day 0**				**Day 7**			
	**MRS**	**MAC**	**EMB**	**NA**	**MRS**	**MAC**	**EMB**	**NA**
A1	1.4 × 10^7^	—	—	—	3.0 × 10^1^	—	—	—
A2	1.2 × 10^1^	—	—	—	5.0 × 10^1^	—	—	—
A3	1.8 × 10^1^	—	—	—	2.0 × 10^1^	—	—	—
A4	1.7 × 10^7^	—	—	—	—	—	—	—
B1	2.2 × 10^7^	—	—	—	2.0 × 10^1^	—	—	—
B2	2.1 × 10^7^	—	—	—	—	—	—	—
B3	1.8 × 10^7^	—	—	—	3.0 × 10^1^	—	—	—
B4	1.9 × 10^7^	—	—	—	6.0 × 10^1^	—	—	—
C1	2.1 × 10^7^	—	—	—	2.0 × 10^1^	—	—	—
C2	2.0 × 10^7^	—	—	—	3.0 × 10^1^	—	—	—
C3	2.2 × 10^7^	—	—	—	2.0 × 10^1^	—	—	—
C4	1.6 × 10^7^	—	—	—	2.0 × 10^1^	—	—	—

Abbreviations: **—:** no growth; A: milk = 70%, coconut = 30%; A1: A + 1.5%LAB; A2: A + 1.5%C.S; A3: A + 1.5%EPS; A4: A; B1: 100%milk + 1.5%LAB + 1.5%C.S; B2: 90%milk + 10%coconut + 1.5%LAB + 1.5%C.S; B3: 80%milk + 20%coconut + 1.5%LAB + 1.5%C.S; B4: 70%milk + 30%coconut + 1.5%LAB + 1.5%C.S; C1: A + 1.5%LAB + 1.5%C.S + 0.5%EPS; C2: A + 1.5%LAB + 1.5%C.S + 1.0%EPS; C3: A + 1.5%LAB + 1.5%C.S + 1.5%EPS; C4: A + 1.5%LAB + 1.5%C.S + 2.0%EPS.

## Data Availability

The data that support the findings of this study are available on request.
